# A survey of poison control centers worldwide

**DOI:** 10.1186/2008-2231-20-13

**Published:** 2012-08-28

**Authors:** Ali Pourmand, Justin Wang, Maryann Mazer

**Affiliations:** 1 Department of Emergency Medicine, George Washington University, Medical Center, Washington, DC, 20037, USA

## Abstract

To stem the rising incidence of toxic exposure as well as the associated morbidity and mortality, the past century has seen the establishment and evolution of poison control centers (PCCs) worldwide. Depending on the location, PCCs vary in terms of staffing model, services offered, and funding sources. In this article, we discuss a survey of poison control centers worldwide.

## Background

Poisonings are a major cause of morbidity and mortality worldwide and as such represent a major public health threat, especially to children, according to a World Health Organization (WHO) report [1]. Potentially toxic exposures can be pharmaceutical, chemical, occupational, or environmental in nature. Technological progress has hastened the rate at which novel chemicals are developed and made available to the public, further raising the potential for adverse effects and outcomes. Our ability to detect chemicals in the body and the environment has increased substantially over the past two decades; however, there is still a paucity of toxicology data due to a lack of controlled data. According to the Centers for Disease Control and Prevention, deaths due to poisoning surpassed deaths related to motor vehicle accidents in the United States in 2008, and this trend appears to be continuing [2].

To stem the rising incidence of toxic exposure as well as the associated morbidity and mortality, the past century has seen the establishment and evolution of poison control centers (PCCs) worldwide. Depending on the location, PCCs vary in terms of staffing model, services offered, and funding sources. Numerous studies have demonstrated the cost-effectiveness of PCCs in terms of number of emergency department visits avoided and decreased hospital length of stay [3-5]. In fact, an assessment by the Institute of Medicine attributes $10 of potential healthcare spending avoided for every $1 spent on funding PCCs [6]. Additionally, the utilization of PCCs has been associated with improved patient outcomes [3-5].

## United States

In the United States, the American Association of Poison Control Centers (AAPCC) oversees the nation’s 57 PCCs by providing accreditation as well as staff certification and continuing education. Healthcare professionals and the general public alike may solicit advice free of charge from PCCs 24 hours a day, 365 days a year, through a national telephone hotline [7]. PCC staff typically include nurses and pharmacists working under the supervision of a board-certified medical toxicologist. The certification process for PCC staff involves logging at least 2,000 telephone encounter hours within a 12-month period and passing a standardized examination. Certified poison information specialists are also required to perform continuing education activities as well as take a re-certification exam every 7 years [8]. The AAPCC also operates the National Poison Data System (NPDS), a web-based software, which allows PCCs to upload information from telephone encounters in near real-time, thereby functioning as a centralized database of exposures as well as health outcomes [7,8]. This serves as a valuable surveillance tool for identifying emerging public health threats (e.g., drugs of abuse, medication and consumer product adverse effects, bioterrorism) on a local, regional, and national scale. Data is also available for epidemiologic research, and the AAPCC publishes an annual report of exposures. Furthermore, the NPDS contains a searchable compendium of toxicologic information on approximately 390,000 chemicals, accessible by PCC staff [8].

The nation’s 57 non-profit PCCs receive approximately 80 % of their funding from state disbursements and the remainder from federal coffers, and occasionally specialized contracts [9]. Budget cuts, however, threaten a reduction in staffing, services, and even the number of PCCs in operation [9]. In fact, of the previously 60 PCCs in operation, 3 closed in 2010 [8]. In 2011, federal expenditure on PCCs decreased by 25 % alongside state-level cuts [10]. The full magnitude of effect has yet to be determined, but it is likely that monetary concerns will have a significant impact on the practice of medical toxicology in the United States.

## Sweden

The Swedish Poisons Information Centre (SPIC), operating out of Karolinska University Hospital, serves as the nation’s sole PCC and is staffed by pharmacists. SPIC pharmacists field queries from the national telephone hotline, accessible 24 hours daily by healthcare professionals and laypersons. These pharmacists receive a minimum of 3 months of in-service training prior to managing telephone encounters independently. Physicians from several specialties, including toxicologists, intensivists, and anesthesiologists, provide medical supervision. The SPIC operates under the auspices of Sweden’s Medical Product Agency and therefore receives funding from the national government [11].

## Japan

In Japan, toxicology has only emerged as a public health priority in recent decades [12]. Founded in 1986, the relatively nascent Japan Poison Information Center (JPIC) operates two PCCs. Healthcare professionals and the lay public may contact the Osaka PCC via telephone 24 hours daily and the Tsukuba PCC during regular business hours (i.e., 0900 to 1700). Pharmacists with post-graduate training in poison information staff each PCC. The centers currently rely on affiliations with local hospitals for medical support from physicians. Despite receiving grants from Japan’s Ministry of Health and Welfare, the Osaka PCC has struggled to meet the demand of its telephone service due to budget constraints and has therefore established priority telephone lines for financial supporters, including private individuals, healthcare professionals, hospitals, and industrial entities. The Ministry of Health and Welfare also supports the JPIC in the development and maintenance of a computerized database of poison information [13].

## Brazil

Brazil’s 36 regional PCCs, in operation 24 hours daily, provide telephone consultations to healthcare professionals and the general public. The National Pharmaco-Toxicological Information System provides poison information support to the PCCs. Brazil also boasts a rich academic climate fostered by the activities of the Brazilian Society of Toxicology, which hosts a biennial Brazilian Congress of Toxicology and promotes a myriad of toxicology training opportunities ranging from certificate programs to doctoral degrees. As PCCs do not technically belong to Brazil’s public healthcare system, they do not receive funding from the national government, and hence rely on volunteer staff and a mélange of precarious funding sources, including local governments as well as affiliated hospitals and universities [5,12].

## United Kingdom

The United Kingdom’s National Poisons Information Service (NPIS) operates 24 hours daily through 4 PCCs [14]. Only registered healthcare professionals may access NPIS programs, which include a telephone service and a web-based poison information database, TOXBASE [14]. The general public may not directly access NPIS programs; rather, laypersons may make inquiries to the National Health Service via a telephone service staffed by nurses with access to TOXBASE. Commissioned by the national Health Protection Agency (HPA), the NPIS receives funding primarily from government grants, but also generates some revenue through research grants and the sale of TOXBASE subscriptions to commercial entities approved by the HPA. NPIS staff maintain TOXBASE, ensuring that its 14,000 product entries provide healthcare professionals with up-to-date information to accurately diagnose and to appropriately manage patient exposures [14]. Furthermore, TOXBASE serves as a training platform for medical schools as well as healthcare professionals seeking continuing education [1].

## Iran

Iran currently operates 29 PCCs, or Drug and Poison Information Centers (DPICs), with the support of its medical universities and the Food and Drug Organization [12]. DPICs respond via telephone, email, or fax to poison information requests from healthcare professionals and laypersons [15]. Qualified responders at DPICs include physicians and pharmacists trained in toxicology. DPICs also disseminate poison information through literature publication and distribution, mass media campaigns, seminars, and other continuing education opportunities [12,15]. Healthcare professionals and the general public alike may get advice free of charge from a telephone hotline available in all parts of the country, 24 hours a day, 365 days a year, with calls routed to the nearest DPIC. In addition to DPICs, Iran has seen the development of numerous poison treatment centers (PTCs) [16]. Typically based out of teaching hospitals, PTCs are specialized in the management of clinical toxicology and serve as repositories for antidotes [17], although the majority of Iran’s emergency departments and intensive care units also routinely handle cases of poisoning [18]. In some instances, PTCs focus on exposures endemic to a particular region, such as Razi Hospital’s expertise in scorpion and snake toxins in southern Iran [16].

## Future directions

The interconnectedness of the global community begs the need for greater collaboration in creating a consolidated, international poison information database with standardized treatment protocols. One of the major limitations of the individual databases is that there is often a paucity of data on foreign products, which will be an emerging concern in a time of increasing global commerce. The International Programme on Chemical Safety, a joint effort by the WHO, the International Labour Organization, and the United Nations Environment Programme, in cooperation with the Canadian Centre for Occupational Health and Safety, currently maintains a web-based resource, INCHEM, which provides peer-reviewed chemical monographs to the public at no charge. INCHEM, however, relies primarily on contributions from Europe, such as the Commission of the European Union, the Swedish Criteria Group, the Nordic Expert Group, and the United Kingdom’s NPIS, and conspicuously lacks the voluminous data amassed in the United States’ NPDS [19]. A comprehensive patient encounter database would equip PCCs worldwide with an enhanced surveillance mechanism to more readily recognize public health threats, such as emerging drugs of abuse (e.g., bath salts, synthetic marijuana) [20]. Moreover, as some PCCs face challenges in obtaining chemical profiles and monographs from industry and manufacturing entities reluctant to voluntarily divulge proprietary information, such as in Japan, the aforementioned organizations should leverage their international influence to foster greater knowledge sharing [13].

With the emergence of mobile technology and social media, PCCs should utilize these platforms for mass marketing to more effectively disseminate public health education and warnings. Additionally, mobile technology may facilitate bringing critical poison information to the bedside clinician.

PCCs worldwide struggle to secure stable sources of funding, with most relying on a combination of governmental support, grants, fundraising, and miscellaneous contract income and subscription fees. Even in times of fiscal austerity, governments should prioritize financial support for PCCs, given the cost savings and improved outcomes they provide.

## Competing interests

The authors have no commercial associations or sources of support that might pose a conflict of interest.

## Authors’ contributions

All authors have made substantive contributions to the study, and all authors endorse the data and conclusions. All authors read and approved the final manuscript.
